# Correlation between Processing Parameters and Degradation of Different Polylactide Grades during Twin-Screw Extrusion

**DOI:** 10.3390/polym12061333

**Published:** 2020-06-11

**Authors:** Olga Mysiukiewicz, Mateusz Barczewski, Katarzyna Skórczewska, Danuta Matykiewicz

**Affiliations:** 1 Institute of Materials Technology, Poznan University of Technology, Piotrowo 3, 61-138 Poznan, Poland; danuta.matykiewicz@put.poznan.pl; 2 Faculty of Chemical Technology and Engineering, UTP University of Science and Technology, Seminaryjna 3, 85-326 Bydgoszcz, Poland; katarzyna.skorczewska@utp.edu.pl

**Keywords:** polylactide, extrusion, degradation, processing parameters

## Abstract

This article presents the effect of twin-screw extrusion processing parameters, including temperature and rotational speed of screws, on the structure and properties of four grades of polylactide (PLA). To evaluate the critical processing parameters for PLA and the possibilities for oxidative and thermomechanical degradation, Fourier-transform infrared spectroscopy (FT-IR), oscillatory rheological analysis, and differential scanning calorimetry (DSC) measurements were used. The influence of degradation induced by processing temperature and high shearing conditions on the quality of the biodegradable polyesters with different melt flow indexes (MFIs)was investigated by color analysis within the CIELab scale. The presented results indicate that considering the high-temperature processing of PLA, the high mass flow index and low viscosity of the polymer reduce its time of residence in the plastifying unit and therefore limit discoloration and reduction of molecular weight due to the degradation process during melt mixing, whereas the initial molecular weight of the polymer is not an essential factor.

## 1. Introduction

In an era when plastic waste disposal is a significant problem worldwide and environmental issues are becoming more and more important to consumers, finding ecological alternatives for conventional materials is indispensable. Because of this, researchers and manufacturers are working to incorporate sustainable, bio-based, and (hopefully) biodegradable polymers into industrial processes. Thanks to their endeavors, global bio-plastic production capacity reached 2.11 million tons in 2019 and is still growing [1]. Although a wide array of available sustainable materials can be successfully utilized, for example thermoplastic starch [2,3], poly(butylene succinate) [4,5], poly(hydroxybutyrate) [6,7], and more [8,9,10], poly(lactic acid), also known as polylactide (PLA) deserves special attention. This bio-based aliphatic polyester is composed of lactic acid (2-hydroxy propionic acid) monomers, which can be obtained by bacterial fermentation from renewable sources such as corn, potatoes, or sugar beets [11,12,13]. A special advantage is that PLA is susceptible to biodegradation and hydrolysis, thanks to which it can be industrially composted [11,14]. As biodegradation of PLA does not occur spontaneously (i.e., specified conditions including temperature and humidity must be ensured [11,15]), this thermoplastic polymer may be successfully re-processed. It should also be noted that polylactide, unlike some bio-plastics, shows advantageous mechanical properties similar to those of polystyrene or poly(ethylene terephthalate) and can be processed using conventional machinery.

All of the factors mentioned above, in addition to relatively low prices (around 2 US$/kg), make polylactide a highly attractive material for many branches of industry. While utilization of PLA used to be limited to medical applications, nowadays different branches of industry use this polymer to manufacture various products, including food packages, fibers and textiles, mulch films used in agriculture, automotive parts, 3D printing filaments, toys, and novelty items, among others [11,16,17]. In order to produce those goods, different processing techniques can be implemented. Injection molding and extrusion are among the most popular ways to process polylactide [12]. Although these are efficient ways to manufacture ready-to-use parts characterized by good mechanical properties and aesthetics, processing conditions such as high temperatures and shearing may result in degradation of the thermoplastic polymer. Various mechanisms of thermal degradation have been proposed, including hydrolysis, radical reactions, random chain scission, depolymerization, or intermolecular transesterification [11,18]. The rate of degradation is influenced by the polymer’s morphology and structure, i.e., molecular weight, polydispersity, stereoisomeric content, and crystallinity degree [13,19,20]. Independent of the phenomena behind this process, thermomechanical degradation of PLA is indicated by a reduction of the polymer’s molecular weight and consequent deterioration of mechanical properties, changes in rheological and crystallization behaviors, and yellowing [11]. As those changes may significantly decrease the quality of the produced goods, it is crucial to determine the influence of processing parameters on the properties of PLA and to evaluate the risk of degradation. Among the most distinct degradation mechanisms causing changes to the properties and quality of final PLA products (excluding hydrolytic degradation resulting from processing conditions as well as weathering and different environmental issues [21]) is thermo-oxidative degradation [22,23,24]. According to Cuadri et al. [23], when a biodegradable polymer is subjected to shear stress and is additionally exposed to elevated temperature in an oxidative atmosphere, the degradation process is intensified, and can be measured with the relative modification index (RMI).

Different investigators have performed studies evaluating PLA properties and structural changes induced by various processing technologies and external factors. Żenkiewicz et al. [25] evaluated mechanical and thermal properties changes of PLA subjected to multiple twin-screw extrusion conducted with constant processing parameters. During the tests, the manufacturer’s recommended processing parameters were used. The results of the tests clearly show that repeated processing results in a significant deterioration of the polylactide performance. The increase in the degree of crystallinity accompanying subsequent processing, as well as the increased MFI value, clearly indicated the degradation of the material and the defragmentation of the polymer chain. Signori and coworkers [26] studied the effects of pre-drying the polymer material as well as temperature on changes in the molecular weight of PLA processed using a Brabender plastograph. They showed that in the case of PLA processing at higher temperatures, the process of thermo-oxidative degradation rather than hydrolytic degradation had a more significant impact on structural polymer changes. In another work, Taubner and Shishoo [27] investigated the effect of twin-screw extrusion process parameters on poly(l-lactide) (PLLA) properties. In their work, the impact of process temperature (210 and 240 °C) and screw rotational speed (20 and 120 rpm) was examined. It has been shown that a longer residence time of the polymer in the plastifying system has a much more significant impact on changes in its molecular weight than the temperature itself. The application of excessively low rotation speeds of the screws significantly contributes to the thermal degradation of PLLA, and even the presence of moisture does not lead to further reduction of the molecular weight.

The solution to the problem of degradation of biodegradable polyesters sensitive to hydrolytic and thermo-oxidative degradation during melting can be reduced by introducing chain extenders [28,29], or in the case of PLA-based composites, surface modification of fillers that usually increase the intensity of hydrolytic degradation [30,31]. However, it should be remembered that PLA crosslinking can strongly suppress biodegradability after its life cycle [32,33].

Polylactide is more and more commonly used in the production of composites reinforced with lignocellulosic fillers of natural origin [34,35,36,37] as well as in the production of blends with different polymers characterized by a significantly higher recommended processing temperature [38,39,40] or in contrast those more susceptible to thermal degradation [41,42]. The most commonly used technology for manufacturing biopolymers and their composites at an industrial scale is co-rotating twin-screw extrusion, due to its high throughput and mixing efficiency [43,44]. Therefore, it is essential to determine the potential ranges of use of different grades of PLA whose rheological properties and structure depend on the intended application without risk of degradation. It is necessary to elucidate the impact of both the process temperature and the rotational speed of the screws, which determine the exposure time of PLA to elevated temperature in the oxidizing atmosphere. Therefore, the aim of this paper is an evaluation of the extrusion processing parameters and thermomechanical degradation relationship of unmodified polylactides. The results of this paper are intended to provide a guideline for researchers and manufacturers who need a fast and easy way to evaluate and predict the possibilities of degradation of PLA.

## 2. Materials and Methods

### 2.1. Materials

In order to find a correlation between the polymer’s structure and susceptibility to degradation, different grades of PLA were chosen as the objects of this investigation, namely: Ingeo^™^ Biopolymer 2500HP, Ingeo^™^ Biopolymer 3100HP, Ingeo^™^ Biopolymer 3260HP, and Ingeo^™^ Biopolymer 4032D, all of which were supplied by Nature Works (Minnetonka, MN, USA). The chosen properties of the resins are presented in Table 1.

### 2.2. Sample Preparation

**Figure 1:**
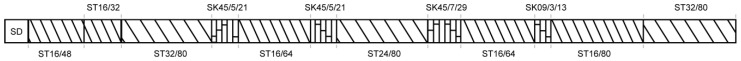
Screw configuration used in the study. The first number denotes the length of a single screw path, and the second number the length of a single segment.

Prior to the extrusion process, the samples were dried in a Memmert ULE 500 laboratory cabinet dryer (Schwabach, Germany) at a temperature of 50 °C for 24 h. The processing of the PLA was conducted using a ZAMAK EH-16.2D co-rotating twin-screw extruder (Skawina, Poland), with screw diameter D = 16 mm and length to diameter ratio L/D = 40, and a die head with capillary die with a diameter of d = 8 mm. The configuration of the screws is presented in Figure 1 (SD: dosing section; SK: kneading section; ST: transporting section), and was previously described in [45]. The maximum processing temperature and the screw’s rotational speed were chosen as the variables. Applied values of the processing parameters and their combinations are collected in Table 2. The extrudates were cooled in the air. The temperature profiles along the plastifying unit are collectively presented in Table 3.

Films needed for the Fourier-transform infrared spectroscopy (FT-IR) measurement and color evaluation were prepared using the compression molding method from the extrudates and from the unprocessed granulates. Firstly, the resin was dried in the Memmert ULE500 laboratory cabinet dryer at a temperature of 50 °C for at least 24 h. Then, the samples were compression-molded between Teflon sheets at a temperature of 180 °C for 5 min using a Remiplast (Czerwonak, Poland) hydraulic press. The resulting thin films with a thickness of c.a. 150 µm were cooled in the air. Compression molding of the samples could contribute to the overall degradation of the material, but it was assumed that application of the relatively low temperature of 180 °C would not significantly alter the sample structures and properties and reduce the differences between the PLA grades extruded in different conditions.

### 2.3. Experimental Methods

Fourier-transform infrared spectroscopy (FT-IR) measurements were obtained by means of a spectrometer Jasco FT/IR-4600 (Tokyo, Japan) at room temperature (23 °C) in attenuated total reflectance (ATR–FT-IR) mode. In total, 64 scans at a resolution of 4 cm^−1^ were used in all cases to record the spectra. The spectra were normalized using the C–H stretching peak at 2995 cm^−1^ as a reference.

Rheological investigations in small-amplitude oscillation shearing mode were carried out on the extruded samples using an Anton Paar MCR 301 rotational rheometer (Graz, Austria) with a 25-mm diameter cone-plate measuring system. The experiments were conducted at 200 °C after drying the specimens for 24 h at 60 °C. The strain sweep experiments were realized before carrying out the dynamic oscillatory frequency sweep measurements in order to determine the linear viscoelastic range (LVE). The strain value determined during the preliminary investigations and used during the frequency sweep experiments was set at 1%, while the frequency range of 0.5 to 500 s^−1^ was used. An evaluation of zero shear viscosity (η_o_) was possible due to the rheological measurements in oscillatory mode and the calculations that were performed applying the Rheoplus 32 v.3.40 software. Zero shear viscosity was determined by the Carreau–Yasuda model fitted to the experimental data. The Carreau–Yasuda model has been described by the following Equation (1):

(1) $$\eta \left(\dot{\gamma }\right)=\eta_{0}{\left[1+{\left(\lambda \dot{\gamma }\right)}^{a}\right]}^{\frac{n-1}{a}}$$

where η_0_ is zero-shear viscosity, n is a power-law coefficient, a is the adjustable exponent (equal to 2 for the simple Carreau model), $\dot{\gamma }$ is a shear rate, and λ is characteristic time [46].

The indirect information on changes in the molecular weight (M_w_) of PLA subjected to extrusion with various processing parameters was evaluated based on rheological data according to Equation (2):

(2) $$\log\eta_{0}=3.4\log M_{w}-C\left(T\right)$$

where η_0_ is the zero shear viscosity calculated by the fitting of Equation (1) to experimental data from oscillatory rheological measurements, and C(T) = 14.83, as revealed from literature data and our preliminary research [47,48].

Differential scanning calorimetry (DSC) measurements, which allow for evaluating the thermal properties of materials, were carried out using a Netzsch DSC 204F1 Phoenix apparatus (Selb, Germany). Material samples of approximately 5.0 ± 0.1 mg were placed in aluminum crucibles with pierced lids, heated up to 210 °C, held in a molten state for 5 min, and cooled back to 20 °C at a rate of 10 °C/min and in an inert nitrogen atmosphere. The heating/cooling cycle was repeated twice to erase the materials’ thermal history and evaluate the DSC curves from the second melting procedure. The crystallization degree (X_c_) was calculated according to Equation (3):

(3) $$X_{c}=\frac{∆H_{m}-∆H_{\mathrm{cc}}}{∆H_{100\%\mathrm{PLA}}}\cdot 100\%$$

where ΔH_m_ is the melting enthalpy of a sample, ΔH_cc_ is the cold crystallization enthalpy of a sample, and ΔH_100%PLA_ is the melting enthalpy corresponding to a 100% crystalline polylactide (93 J/g) [49].

The color of PLA samples was evaluated according to the International Commission on Illumination (CIE) through L*a*b* coordinates [50]. In this system, L* is the color lightness (L* = 0 for black and L* = 100 for white), a* is the green (−)/red (+) axis, and b* is the blue (−)/yellow (+) axis. The color was determined by optical spectroscopy using HunterLab Miniscan MS/S-4000S spectrophotometer (Reston, VA, USA), placed additionally in a specially designed light trap chamber. The total color difference parameter (ΔE) was calculated in relation to a white standard according to Equation (4) [51]:

∆E = [(∆L*)^2^ + (∆a*)^2^ + (∆b*)^2^]^0.5^ (4)

The yellowness index (YI) was determined as presented in Equation (5) according to the ASTM E 313 standard, after recalculation of color parameters to XYZ coordinates:

(5) $$\mathrm{YI}=100\cdot \left(C_{X}X-C_{Z}Z\right)/Y$$

where C_X_ = 1.3013 and C_Z_ = 1.1498 for the D65 CIE standard illuminant and observer.

The thermal stability of all the specimens was evaluated by thermogravimetric analysis (TGA) using a Netzsch TG209 F1 apparatus (Selb, Germany). The 5 ± 0.1 mg samples were placed in Al_2_O_3_ crucibles and heated from 30 °C to 900 °C at a rate of 10 °C/min in the inert nitrogen atmosphere. A dedicated software calculated the first derivative of the obtained TGA curves (dTG).

## 3. Results

### 3.1. FT-IR Spectroscopic Analysis

**Figure 2:**
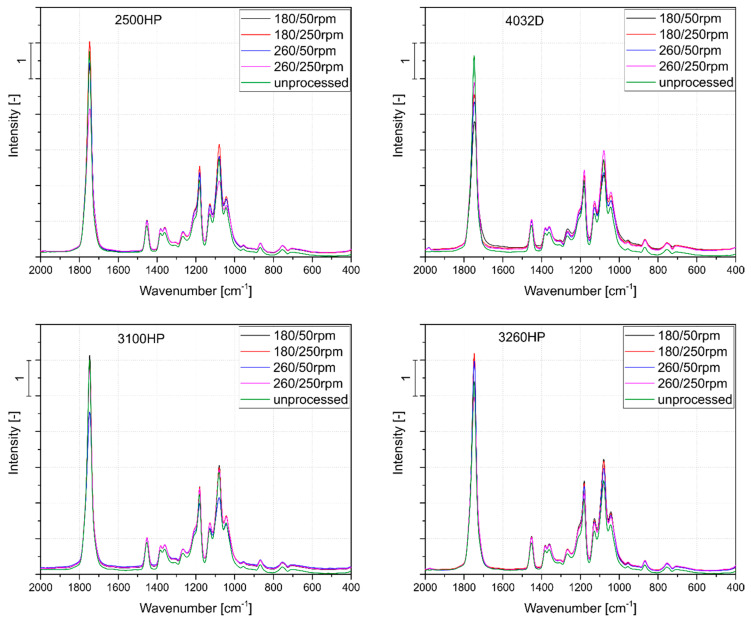
Fourier-transform infrared spectroscopy (FT-IR) spectra of PLA grades processed in an extreme range of processing parameters, as well as the compression-molded unprocessed materials.

Figure 2 presents a summary of absorption spectra for four grades of polylactide processed at extreme (i.e., the highest or the lowest) parameters of temperature and screw rotational speed as well as the unprocessed materials. It should be emphasized that no additional peak was observed for any of the materials due to the applied processing conditions, but there was a change in the intensity of the peaks that were characteristic for PLA. The peaks observed in the spectra of the processed samples were comparable with the compression-molded granulates. For all the tested materials, the most pronounced changes were noted for the 1750 cm^−1^ band, corresponding to carbonyl group stretching. Moreover, significant changes in peak intensities at 1085 cm^−1^ and 1183 cm^−1^ (asymmetric vibration of ester group) were observed, which were associated with thermo-oxidative and/or thermomechanical degradation resulting in random PLA chain scission and the phenomenon of formation of anhydrides, carbonyl, and carboxyl groups [23,52,53]. For all the polylactide varieties used, the processing with a lower speed of screws resulted in a significant reduction in the intensity of the absorption band at 1750 cm^−1^. This was especially evident in the case of the crystalline polylactide varieties. The 4032D polymer showed a relatively small change in the intensity of the observed peaks, which may indicate a similar degree of degradation (or lack thereof) of the polymer for all the applied parameter sets. For polymers with a high MFI value, significant changes in the case of FT-IR spectra in the range of 2000–400 cm^−1^ were noted only for the material series processed at the highest processing temperature and the lowest screw speed (260 °C and 50 rpm). Therefore, it can be concluded that materials with lower viscosities, due to their rheological properties, can be subjected to higher extrusion throughputs and associated shorter material residence times at high temperatures. The processing of the materials did not affect the crystalline structure of the PLA sufficiently to assess the semi-crystalline structure during the forming of thin films by compression molding. Usually, the presence of PLA crystalline domains may be observed as absorption bands at 920 cm^−1^ and 1207 cm^−1^, resulting from flexural C-H bond vibration and alkyl-ketone chain vibration, respectively. In the case of tested samples, no significant changes were noted at the mentioned wavelengths. Therefore, it may be concluded that thermo-oxidative and thermo-mechanical degradation of PLAs was not sufficient to create high amounts of low molecular weight polymeric chains able to crystallize in used cooling conditions [54].

### 3.2. Rheological Evaluation of PLA Degradation

**Figure 3:**
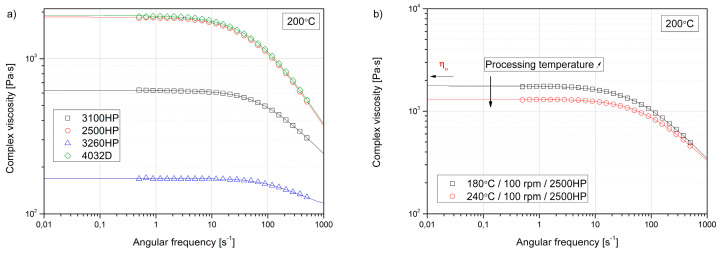
Complex viscosity curves of unprocessed PLA grades (**a**), and a comparison of the influence of processing temperatures on the complex viscosity curve of grade 2500HP (**b**).

Figure 3a shows the complex viscosity curves of four unprocessed polylactide grades tested in the same measuring conditions. The Carreau–Yasuda model was fitted to the experimental data. It can be seen that the high MFI grades showed significantly lower viscosities in the whole angular frequency range. The higher the MFI of the PLA, the more distinct the observed Newtonian flow region. The measured difference of this range between grades 2500HP and 4032D, characterized by similar rheological properties and high flow injection molding of grade 3260HP, was one order of magnitude at 2 s^−1^ and 20 s^−1^, respectively.

All the polymers showed a distinct Newtonian flow plateau. Therefore, the assessment of the zero-shear viscosity based on the Carreau–Yasuda model could proceed. This analysis was important because the rheological measurements carried out in the range of low values of angular frequency allowed for an indirect assessment of degradation changes of thermoplastic polymer materials. During polymer degradation as a result of significant thermal or thermo-mechanical loads, chain scission phenomenon usually occurs [23,55]. As the degree of degradation increases, the differences in the measured viscosities are significantly increased [23], especially in the range of low angular frequency values, usually observed for most of the thermoplastic polymers as the Newtonian flow range. An example of the effect of partial thermal degradation of PLA 2500HP on changes in rheological properties assessed in the range of low values of angular frequency is shown in Figure 3b. Increasing the temperature of the extrusion process from 180 °C to 240 °C caused a decrease in the viscosity value by about 500 Pa·s.

The assessment of the impact of the parameters of the technological process of twin-screw extrusion on the degree of degradation of selected polylactide varieties was based on a comparison of the decrease in the zero-shear viscosity value. The zero-shear viscosity values of tested PLA grades as a function of the processing temperature and rotational speed used during twin-screw extrusion are presented in Table 4. Additionally, the values of characteristic relaxation time λ calculated from Equation (1) related to the onset of non-Newtonian or shear thinning behavior was evaluated [56]. Based on the analysis of the results of rheological measurements, it can be stated that all PLA varieties did not undergo significant degradation processes due to processing in the temperature range up to 200 °C. In the case of PLA varieties with lower melt flow indexes, i.e., 4032D and 2500HP, a decrease in the η_o_ value at 220 °C could be observed. For all the tested materials, the use of the highest processing temperature, i.e., 260 °C, resulted in a significant decrease in the zero-viscosity value. It should be added that in the case of PLA 3100HP and 3260HP, the dependence of the change of η_o_ noted at the highest process temperature was dependent on the rotational speed of the screw. In the case of materials with a higher flow rate, increasing the rotational speed reduced the residence time of the polymer material in the plastifying unit, resulting in reduced polymer degradation. The results are in a good agreement to those presented by Cuadri et al. [23], in which the distinct degradation of the PLA was observed for the samples subjected to melt processing in shearing conditions above 200 °C.

**Figure 4:**
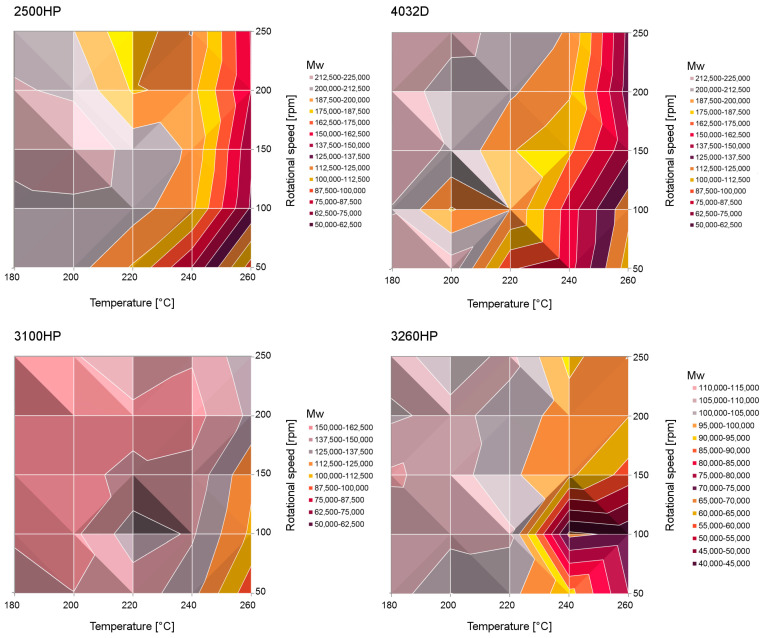
Molecular weights calculated from the rheological data of processed PLA.

It was found that the higher the rotational speed of the screws, the smaller the decrease in the viscosity of polymers. The changes of the zero-shear viscosity of the samples as a function of extrusion temperature and the rotational speed of the screws are presented in Figure 4. The overall degradation of processed materials in a non-inert atmosphere was mostly caused by thermo-oxidative degradation. The thermo-mechanical degradation consists of the effect of both shearing and temperature on the polymeric matrix. The extended exposition to thermal stress caused the additional thermomechanical degradation effects on the PLA matrix. Although processing the polylactide samples with a high rotational speed of the screws caused an increase of shearing, this effect was compensated by shorter processing time; therefore, the effects of degradation (i.e., a decrease of viscosity) were limited. The highest resistance to degradation caused by improperly selected processing conditions, i.e., high process temperature and rotational speed of screws, was shown by the polylactide variety marked as 3100HP. Based on the conducted research, it can be stated that the use of the assessment of the change in the theoretical value of the viscosity determined at zero shear rate can be a useful criterion for assessing polylactide degradation, and is much more sensitive than spectroscopic analysis. Yu and Wilkes discussed the influence of molecular weight distribution of polyethylene on melt relaxation behavior of polyethylene [56]. According to their studies, it can be stated that the higher values of characteristic relaxation time λ, determined from Carreau–Yasuda model fitting to experimental data, suggest the broader molecular weight distribution. The characteristic relaxation time is directly correlated with longer molecular relaxation time and shows the influence of the higher molecular weight of the main chain of polymers. This finding is in line with the results obtained for PLA grades processed in different conditions. 3260HP was defined as the polymer with the lowest polydispersity (Table 1), which resulted in the lowest values of λ. However, it should be underlined that the temperature/rotational speed range causing the highest degradation causes much higher relaxation time values than for the remaining materials, which suggests the creation of a higher amount of PLA chains with lower molecular weight. The higher temperature and longer residence in elevated conditions of the polymer were shown by the higher values of λ, confirming the degradation of the polymer. This effect was especially pronounced for PLA grade 4032D.

The molecular weights calculated according to Formula (2) using the rheological data of various processed PLA grades are presented in Figure 4. Although the molecular weight values discussed here were estimated based on the theoretical principles and were not determined experimentally, they can be used to analyze relative changes between the polymer processed in different conditions. In addition, the theoretical relationship of zero shear viscosity is generally recognized as a reliable one [48]. It can be observed that all the M_w_ values differ from the initial ones collected in Table 1. It should be noted that the slightly higher values obtained for the processed grades in comparison to the as-received ones did not result from changes in their molecular structures, but rather from a slight discrepancy between the theoretical model and the real materials. In the case of processing at 180 °C, all the tested PLA grades showed the highest molecular weight independently of the screw’s rotational speed. The lowest M_w_ was reported for the samples processed at 260 °C, 50 rpm. The exposition of PLA to elevated temperature in oxidative conditions caused the chain scission mechanism of alkoxyl radicals, resulting in the development of novel free radicals and subsequent further chain scission of polymeric chains [24,52]. Interestingly, even though the initial molecular weight differed notably among the tested grades (i.e., from 107,500 Da for 3260HP to 202,000 Da for 4032D), the minimum values were quite similar, in the range of 75,000 Da for 260 °C/50 rpm/3260HP to 106,400 Da for 260 °C/50 rpm/4032D. It could be seen that the high-MFI PLA grade 3100HP showed the smallest molecular weight variation in the full range of considered processing parameters. A relatively small influence of process parameters was noted. Similarly, based on the obtained results it can be stated that the high-crystalline PLA grade 2500HP could be processed in a temperature range up to 220 °C without a significant reduction of M_w_. In the case of the material with the highest MFI value (3260HP), degradation changes in the range above 220 °C and low rotational speeds could be observed. Therefore, the studied materials can be processed with a wide range of parameters, which enables them to be used for the production of blends of materials with high melting temperatures.

### 3.3. Calorimetric Investigation of PLA Morphology Change

Data on the thermal parameters obtained for different polylactide grades processed under different conditions and characteristic phenomena such as glass transition temperature (T_g_), melting temperature (T_m_), and crystallinity (X_c_) calculated according to Equation (3) are presented collectively in Table 5. In the case of cold crystallization temperature, no clear relationship was found between the extrusion parameters of the polymer and its value; therefore, the evaluation of cold crystallization was not taken into consideration in the analysis.

For polylactide grade 2500HP processed at variable temperatures and screw rotational speeds, the glass transition temperature occurred in the range from 59.4 °C to 62.1 °C, with the lowest value recorded for the combination of parameters 260 °C/50 rpm and the highest for the combination 180 °C/200 rpm. It can be stated that the T_g_ value decreased as the processing temperature increased. According to Rasselet and coworkers [57], it is known that a decrease in T_g_ may be related to the chain scission process occurring as an effect of oxidation. The DSC data confirm the lowered molecular weight determined by rheological evaluation. The relationship between the processing temperature and the T_m_ value is similar. The highest melting point, equal to 179.5 °C, was recorded for the 200 °C/150 rpm/2500HP sample and the lowest, equal to 172.2 °C, was recorded for the 260 °C/100 rpm/2500HP sample. The degree of crystallinity of the tested samples of 2500HP was at a similar level, ranging from about 16% to 23%. The sample processed at 200 °C and 150 rpm showed the lowest degree of crystallinity equal to 16.0%.

The 3100HP polylactide samples exhibited glass transition temperature values from 59.6 °C to 61.7 °C. The lowest value was observed for the combination of parameters 260 °C/150 rpm, and the highest for the combination of 240 °C/250 rpm. The T_g_ of the remaining samples showed intermediate values; no relationship was observed between extrusion parameters and glass transition. Similarly to the 2500HP variety, it could be seen that the melting point decreased as the processing temperature increased, with the highest T_m_ value being 176.2 °C for sample 180 °C/150 rpm/3100HP and the lowest T_m_ value being 172.4 °C for sample 260 °C/50 rpm/3100HP. The degree of crystallinity of all samples based on the 3100HP polymer exceeded 20%, with the lowest value being shown by the material extruded at 260 °C at a rotational speed of 100 rpm. The 200 °C/250 rpm/3100HP sample showed the highest X_c_ value of 27.3%.

The glass transition temperature of most samples based on 3260HP was around 60 °C, with the exception of sample 200 °C/200 rpm/3260HP, for which the T_g_ was 58.2 °C and 240 °C/200 rpm/3260HP, where the glass transition occurred at 61.4 °C. The melting point of the crystalline phase of the samples tested dropped as a function of extrusion temperature. The highest T_m_ value of 176.5 °C was observed for the processed material with the combination of 200 °C/100 rpm, and the lowest T_m_ of 174.5 °C was obtained with a combination of 260 °C/50 rpm. The degree of crystallinity of the samples varied in the range of 21.0–29.7%, with extreme values recorded for the samples at 180 °C/100 rpm and 180 °C/50 rpm, respectively.

The glass transition of 4032D samples occurred in the range of 59.7 °C (for sample 260 °C/100 rpm/4032D) to 61.9 °C (for sample 180 °C/200 rpm/4032D). No relationship was observed between the processing conditions and the T_g_ value. For several 4032D PLA samples, a double melting temperature peak was observed. A maximum of the endothermic peak was distinguished at a temperature of about 165 °C and a temperature of 170 °C. A similar phenomenon was described by Magoń and Pyda [58]. During the double melting of the 4032D polymer, the first (low-temperature) peak can be attributed to the melting of imperfect crystals, and the second one to melting of the remaining crystalline fraction [59]. Another possible explanation for this phenomenon is the occurrence of a melting–recrystallization mechanism [60]. The lowest melting temperature values were recorded for samples 260 °C/50 rpm and 260 °C/100 rpm, and the highest for material extruded at 220 °C at a screw speed of 100 rpm. The degree of crystallinity of the 4032D polylactide was lower in comparison to all the tested PLA samples and ranged from 9.4% (for sample 200 °C/250 rpm) to 18.0% (for 180 °C/200 rpm).

It is widely known that the crystallization behavior of PLA depends on its molecular weight and processing conditions [54,61]. Taking into consideration all the gathered data on thermal properties of different polylactide grades extruded at variable temperatures and rotational speeds, it can be noted that the observable changes of T_g_ and T_m_, as well as X_c_, were small considering the resolution of the measuring system. In addition, no direct correlation of the melting/crystallization behavior and the course of temperature and/or speed changes could be found. The results of DSC were confirmed with infrared spectroscopy, which did not show observable changes in PLA crystallinity. Therefore, it can be concluded that processing in temperature up to 260 °C with high shear rates should not notably alter the thermal properties of PLA.

### 3.4. PLA Color Changes Induced by Thermomechanical Conditions of Twin-Screw Extrusion

**Figure 5:**
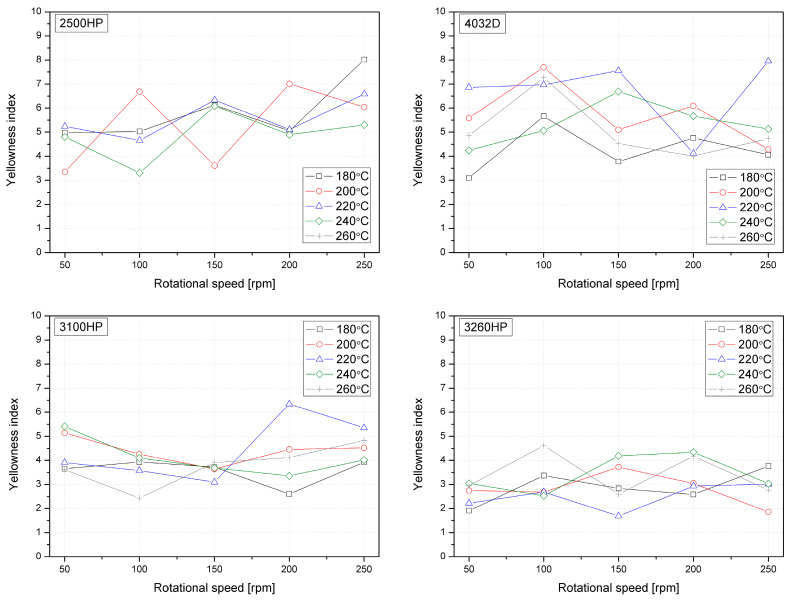
The yellowness index (YI) of PLAs processed with various processing parameters.

Table 6 summarizes the color change measured according to the CIELab system using a wide-angle spectrophotometer of the material samples processed at variable temperatures and rotation speeds of screws. Samples in the form of semi-translucent thick foil were tested on a white sheet of defined color with parameters L* 91.56, and a* 1.83, b* −9.93. A color change usually accompanies intensive thermo-oxidative degradation of thermoplastic polymers [62]. The most characteristic changes are yellowing of the material, indicated by the increase of the parameter b* in the CIELab [63], which, according to Cai [62], is the most accurate colorimetric coordinate for assessment of potential degradation of polymers. Additionally, the yellowness index (YI) was calculated according to Equation (5) for all extruded polymer series and is presented in Figure 5. The lower the YI, the better the color performance, while higher YI values give comparable information about the yellowing of the PLA after processing. On the basis of the performed investigations it can be stated that PLA grades with higher MFI show lowered YI in comparison to those with higher viscosity. The biggest spread of the YI values was observed for amorphous PLA grade 4023D, while both the lowest YI values as well most constrained variations of this characteristic were noted for 3260HP. Based on the conducted tests, it can be concluded that materials with a lower melt flow index value undergo more intense discoloration compared to polylactide varieties with a higher MFI. An increased degree of degradation was noted primarily in the case of more viscous polylactide varieties (2500HP and 4032D); this was due to the longer residence time of the polymer material in the plasticizing system in the case of processing at high temperatures (240 °C and 260 °C), and higher values of shear stress occurring during the plasticizing process of the polymer material at lower temperature settings (180–220 °C) in comparison to high flow grades. The second variable parameter of the technological process, i.e., the rotational speed of the screws, had a significantly smaller impact on the color change of the processed biodegradable polyesters. Therefore, it can be concluded that in the case of more viscous materials, it is important to take this parameter into account when selecting the conditions for the melt mixing process planned for further research related to the modification of selected PLA varieties. The total color difference calculated in accordance with Equation (4) allows us to quantitatively compare the discoloration of the materials caused by various factors [51] in the considered case of thermo-oxidative and thermo-mechanical degradation of the polymer during twin-screw extrusion. According to ISO: 2813 almost all the PLA series show an ΔE parameter below 3.5, which is a higher value describing medium color variation. The lowest susceptibility to discoloration was shown by PLA grade 2500HP, which was not entirely consistent with the results of decreased molecular weight and viscosity. The lowest variation of ΔE parameter was observed for 3100HP PLA series, which if it is be compared to relatively low variations of molecular weight in the considered temperature-rotational speed range is in a good agreement. Therefore, it can be stated that a crucial parameter from a qualitative point of view and with respect to discoloration resistance during melt processing is the MFI (which should be high enough to limit the exposure to elevated temperature in oxidative conditions), in addition to the molecular weight of the processed polymer.

### 3.5. Thermal Stability of Extruded PLA Grades

**Figure 6:**
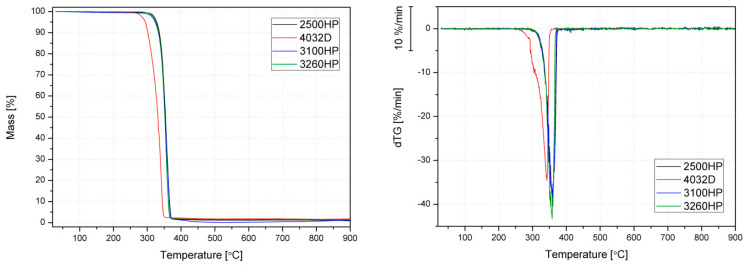
TG (changes of samples’ mass in function of temperature) and the first derivative of the obtained TG curves (dTG) of PLA grades.

Although PLA is made from natural resources, it has relatively high thermal stability. Figure 6 shows TG and dTG curves for the used polymeric materials. Only PLA 4032D series shows the temperature at the beginning of degradation, read from a dTG curve lower than that applied during processing in a molten state. Other PLA grades show a comparable thermal degradation process as assessed by the TGA method. The measurements were carried out in an inert gas atmosphere, and therefore it can be concluded that in most cases, the degradation processes causing the observed structural and qualitative changes in polymers resulted from the phenomenon of thermo-oxidative and thermomechanical degradation. According to the literature reports, in the presence of an oxidizing atmosphere degradation begins at a temperature of about 200 °C [23].

Table 7 presents the temperature values corresponding to 5% and 10% mass loss of the processed PLA samples (T_5%_ and T_10%_, respectively). Unfortunately, it is difficult to determine a clear trend based on the obtained results. Only in the case of significant degradation caused by prolonged exposure to the highest temperature of 260 °C could a comparable decrease in thermal stability for all tested materials be observed. However, the most significant changes in the temperature of the onset of degradation assessed based on T_5%_ and T_10%_ were shown by the amorphous PLA variety with a low MFI (4032D). Interestingly, unlike the results of rheological tests, the 2500HP polymer showed the highest thermal stability as assessed by the TGA method, which may be associated with the high molecular weight of the polymer [64]. The high-MFI 3100HP and 3260HP PLA varieties showed comparable changes in thermal stability.

## 4. Conclusions

The aim of this study was to perform an analysis of the influence of processing parameters on the degradation of different grades of polylactide. The results of infrared spectroscopy, rheological measurements, differential scanning calorimetry, and colorimetry showed that during extrusion at high temperatures (up to 260 °C) and with a high rotational speed of screws the polymer undergoes thermo-mechanical and thermo-oxidative degradation, which results in decreased viscosity, yellowing, and a reduction of molecular weight. The combination of a low rotational speed of screws and a high temperature had the most notable influence on the deterioration of polymer properties. For example, the zero shear viscosity of grade 4032D decreased about 10 times with the increase in temperature from 180 °C to 260 °C at 50 rpm. However, it was found that the PLA grades characterized by low initial viscosity (high melt flow index) were less susceptible to degradation during extrusion with high throughput, mainly because of the shorter residence time in the plastifying unit. Grade 3260HP, characterized by MFI of 65 g/10 min, showed an about 4-fold decrease of zero shear viscosity due to processing, whereas grade 2500HP with MFI of 8 g/10 min experienced a decrease of almost 20 times. Interestingly, no observable difference was noticed between the crystalline and amorphous PLA grades. In addition, the influence of the applied processing conditions on the crystallization behavior of the studies of the samples by DSC was slight. The crystallinity degree of the crystalline grades was about 20–30%, independent of the processing conditions. It can be concluded that even though PLA is known for being sensitive to processing conditions, its less viscous grades can be successfully extruded with high throughputs without a severe deterioration of the material’s quality.

## Figures and Tables

**Table 1:** Chosen properties of the studied polylactide (PLA) grades according to the technical datasheets.

Property	2500HP	3100HP	3260HP	4032D
Mass average molar mass M_w_ (Da)	193,250	148,250	107,500	202,000
Polydispersity index (M_w_/M_n_)	1.85	1.81	1.68	1.80
Density (g/cm^3^)	1.24	1.24	1.24	1.24
Mass flow rate (g/10 min)	8.0	24	65	7.0
Yield strength (MPa)	64 ^a^/65.5 ^c^	65 ^a^/64 ^c^	64 ^a^	60 ^a^
Elongation at break (%)	3.6 ^a^/4.3 ^c^	3.4 ^a^/2.2 ^c^	3.0 ^a^/1.3 ^c^	6.0 ^a^
Izod’s impact strength (J/m)	18.7 ^a^/40 ^c^	16.2 ^a^/32 ^c^	16 ^a^/16 ^c^	16.0 ^a^
Heat deflection temperature (HDT) (°C)	54 ^a^/144 ^c^	54 ^a^/149 ^c^	56 ^a^/151 ^c^	-
Melting temperature (°C)	165–180	165–180	165–180	155–170

^a^—amorphous; ^c^—crystalline.

**Table 2:** Combinations of processing parameters.

Die Temperature (°C)	Screw Rotational Speed (rpm)				
	50	100	150	200	250
180	180/50	180/100	180/150	180/200	180/250
200	200/50	200/100	200/150	200/200	200/250
220	220/50	220/100	220/150	220/200	220/250
240	240/50	240/100	240/150	240/200	240/250
260	260/50	260/100	260/150	260/200	260/250

**Table 3:** Temperature profiles used during extrusion corresponding to die in various maximum temperature setups.

The Temperature of the Heating Zones (°C)								
Die Temperature	1	2	3	4	5	6	7	8
180	180	175	175	170	165	155	150	135
200	195	185	175	170	165	155	150	135
220	215	205	195	195	170	160	150	135
240	230	220	210	195	180	170	150	135
260	250	240	230	210	190	170	160	135

**Table 4:** Zero shear viscosity and characteristic relaxation time.

η_0_ (Pa·s)/λ (ms)						
**2500HP**	**Screw Rotational Speed**	**Temperature**				
		**180 °C**	**200 °C**	**220 °C**	**240 °C**	**260 °C**
	50 rpm	1844/14.5	1707/13.4	1223/9.8	580/37.4	119/18.9
	100 rpm	1765/15.8	1806/13.7	1717/14.8	1312/12.0	414/11.4
	150 rpm	2102/15.8	2305/13.3	1769/15.2	1521/13.0	458/17.7
	200 rpm	1971/13.8	1836/14.8	1223/14.9	1340/11.0	582/18.0
	250 rpm	1740/16.1	1570/13.5	1043/10.4	1582/12.6	573/7.5
**4032D**	**Screw Rotational Speed**	**Temperature**				
		**180 °C**	**200 °C**	**220 °C**	**240 °C**	**260 °C**
	50 rpm	2088/14.5	2162/11.7	790/35.0	651/9.6	183/34.4
	100 rpm	1902/13.9	1229/16.9	1552/16.6	673/36.2	244/41.7
	150 rpm	2132/14.4	1872/14.0	1277/14.7	1040/8.9	478/30.0
	200 rpm	2 295/16.4	1636/16.7	1224/14.5	1290/15.6	374/23.5
	250 rpm	2 166/14.1	2149/13.7	1707/13.2	1542/15.5	397/15.3
**3100HP**	**Screw Rotational Speed**	**Temperature**				
		**180 °C**	**200 °C**	**220 °C**	**240 °C**	**260 °C**
	50 rpm	546/14.6	586/7.7	526/5.2	354/11.5	108/15.3
	100 rpm	621/8.6	622/16.2	364/11.6	455/12.6	204/22.4
	150 rpm	642/8.1	610/8.2	567/25.0	540/11.2	237/66.1
	200 rpm	618/19.0	651/16.3	617/8.7	634/1.9	406/13.7
	250 rpm	723/9.3	598/22.2	509/8.2	572/7.0	354/54.5
**3260HP**	**Screw Rotational Speed**	**Temperature**				
		**180 °C**	**200 °C**	**220 °C**	**240 °C**	**260 °C**
	50 rpm	191/24.8	147/9.8	156/10.5	111/10.7	56/25.8
	100 rpm	182/4.2	184/7.1	180/28.7	141/20.5	47/54.5
	150 rpm	210/4.7	189/12.4	152/2.2	129/60.3	95/57.2
	200 rpm	169/13.6	184/5.0	162/8.2	137/14.5	125/3.7
	250 rpm	187/17.2	158/17.0	183/24.2	117/10.2	149/3.5

**Table 5:** Thermal parameters obtained from differential scanning calorimetry (DSC) analysis: crystallization degree (Xc), glass transition temperature (Tg), and melting temperature (Tm).

X_c_ (%)/T_g_ (°C)/T_m_ (°C)						
**2500HP**	**Screw Rotational Speed**	**Temperature**				
		**180 °C**	**200 °C**	**220 °C**	**240 °C**	**260 °C**
	50 rpm	22.9/61.0/176.3	19.5/61.4/176.3	21.1/61.2/175.7	19.7/59.8/175.0	21.4/59.4/172.4
	100 rpm	17.3/61.2/176.3	19.6/60.1/176.3	17.4/61.2/176.0	22.4/61.0/175.6	23.2/59.8/172.2
	150 rpm	18.7/61.5/176.6	16.0/61.1/179.5	19.2/61.3/176.0	22.3/60.2/175.6	21.7/61.0/174.3
	200 rpm	18.3/62.1/176.7	20.1/60.7/176.1	17.9/61.3/176.0	18.1/60.7/176.1	23.0/59.9/174.4
	250 rpm	20.1/61.8/176.3	20.3/60.6/175.9	20.4/61.2/175.7	20.2/61.0/175.7	22.8/60.4/174.7
**4032D**	**Screw Rotational Speed**	**Temperature**				
		**180 °C**	**200 °C**	**220 °C**	**240 °C**	**260 °C**
	50 rpm	9.9/60.4/169.9	10.9/61.3/170.2	9.6/60.8/$\frac{163.4}{170.0}$	10.2/60.8/$\frac{164.3}{170.2}$	11.7/59.8/168.2
	100 rpm	11.1/60.6/170.0	13.8/59.8/$\frac{164.7}{169.9}$	10.7/60.1/$\frac{165.4}{170.7}$	12.7/60.6/$\frac{164.9}{170.4}$	11.6/59.7/$\frac{162.2}{169.3}$
	150 rpm	13.6/61.3/$\frac{165.2}{170.2}$	9.8/61.0/$\frac{165.1}{170.1}$	12.2/60.6/$\frac{165.5}{170.2}$	14.0/60.8/$\frac{164.9}{170.3}$	9.8/60.1/$\frac{163.5}{170.1}$
	200 rpm	18.3/61.9/170.5	11.0/61.2/170.1	10.3/61.3/$\frac{164.8}{170.3}$	13.7/60.7/$\frac{164.9}{170.1}$	11.6/60.8/$\frac{162.7}{169.7}$
	250 rpm	12.0/60.0/$\frac{165.2}{170.2}$	9.4/60.7/$\frac{165.1}{170.3}$	10.4/60.8/$\frac{165.2}{170.4}$	12.2/61.0/$\frac{164.2}{170.5}$	9.9/60.5/$\frac{163.4}{170.3}$
**3100HP**	**Screw Rotational Speed**	**Temperature**				
		**180 °C**	**200 °C**	**220 °C**	**240 °C**	**260 °C**
	50 rpm	21.4/61.1/176.1	21.5/60.1/175.5	26.4/60.8/175.1	25.0/61.3/174.8	25.4/60.1/172.4
	100 rpm	24.7/61.2/175.8	21.5/61.1/175.8	23.0/60.1/175.5	23.5/60.8/175.2	20.0/60.2/174.5
	150 rpm	20.5/60.3/176.2	20.3/60.4/176.0	23.5/61.0/175.7	22.9/60.7/175.4	22.1/59.6/174.7
	200 rpm	20.2/60.7/175.8	20.5/60.6/175.9	23.7/60.5/175.7	21.3/60.8/176.1	25.0/60.7/175.0
	250 rpm	20.1/60.6/175.6	27.3/61.2/175.5	25.1/60.9/175.7	21.7/61.7/175.9	21.4/60.6/175.3
**3260HP**	**Screw Rotational Speed**	**Temperature**				
		**180 °C**	**200 °C**	**220 °C**	**240 °C**	**260 °C**
	50 rpm	29.7/60.6/176.0	26.5/59.8/175.9	26.1/60.1/175.8	25.8/60.1/175.1	25.0/60.8/174.5
	100 rpm	21.0/59.8/176.4	24.2/60.0/176.5	26.6/60.6/176.2	24.8/60.4/175.5	22.5/58.9/174.6
	150 rpm	27.0/60.4/175.9	26.2/60.7/176.1	26.3/60.2/175.4	25.1/60.0/175.9	24.8/60.5/174.9
	200 rpm	24.7/61.0/176.3	24.7/58.2/176.2	25.0/60.8/176.3	27.0/61.4/175.4	26.1/60.4/175.6
	250 rpm	24.6/60.3/176.3	25.8/60.2/176.3	28.7/60.6/176.2	27.5/60.7/175.5	22.3/60.9/176.0

**Table 6:** L*, a*, b* and ΔE color parameters obtained for PLAs under various processing conditions.

L*/a*/b*/ΔE						
**2500HP**	**Screw Rotational Speed**	**Temperature**				
		**180 °C**	**200 °C**	**220 °C**	**240 °C**	**260 °C**
	50 rpm	89.9/–0.6/2.7/1.4	89.7/–0.5/1.9/0.5	88.4/–0.5/2.8/1.8	89.0/–0.6/2.6/1.2	90.1/–0.3/1.5/0.6
	100 rpm	88.6/–0.4/2.6/1.6	87.2/–0.53.5/3.2	88.3/–0.4/2.5/1.8	89.3/–0.3/1.7/0.8	89.3/–0.5/2.4/0.8
	150 rpm	88.0/–0.5/3.2/2.3	89.3/–0.3/1.9/0.8	87.8/–0.6/3.4/2.7	88.2/–0.6/3.2/2.2	89.5/–0.5/2.1/0.6
	200 rpm	88.4/–0.4/2.6/1.7	87.4/–0.6/3.7/3.1	88.5/–0.4/2.7/1.6	88.6/–0.3/2.5/1.5	88.8/–0.5/3.0/1.6
	250 rpm	86.6/–0.6/4.2/4.1	87.3/–0.5/3.2/3.0	87.4/–0.3/3.4/3.0	88.0/–0.5/2.8/2.2	89.7/–0.2/–1.6/0.7
**4032D**	**Screw Rotational Speed**	**Temperature**				
		**180 °C**	**200 °C**	**220 °C**	**240 °C**	**260 °C**
	50 rpm	89.5/–0.3/1.7/1.2	87.7/–0.4/2.9/2.5	87.5/–0.7/3.7/3.0	89.5/–0.5/2.3/0.8	88.7/–0.5/2.6/0.6
	100 rpm	88.4/–0.6/3.0/1.9	86.5/–0.6/4.0/4.0	87.4/–0.6/1.7/3.0	88.6/–0.5/2.7/1.6	88.5/–0.8/3.9/2.3
	150 rpm	89.0/–0.4/2.0/1.3	88.6/–0.5/2.7/1.6	87.6/–0.7/4.0/3.0	87.9/–0.7/3.6/2.5	89.4/–0.4/2.4/0.8
	200 rpm	88.2/–0.5/2.5/2.0	87.4/–0.7/3.3/2.9	89.1/–0.3/2.2/1.2	88.5/–0.4/2.9/1.7	89.6/–0.4/2.1/0.7
	250 rpm	89.0/–0.5/2.2/1.3	88.7/–0.4/2.3/1.6	87.2/0.6/4.2/3.4	88.3/–0.2/2.6/1.8	89.2/–0.6/2.6/1.0
**3100HP**	**Screw Rotational Speed**	**Temperature**				
		**180 °C**	**200 °C**	**220 °C**	**240 °C**	**260 °C**
	50 rpm	89.3/–0.3/1.9/1.9	88.7/–0.3/2.7/2.8	89.4/–0.4/2.1/1.9	88.4/–0.3/2.8/3.1	90.1/–0.4/2.0/1.3
	100 rpm	89.1/–0.4/2.1/2.1	89.2/–0.4/2.3/2.1	89.2/–0.3/1.9/2.0	88.8/–0.4/2.2/2.4	90.4/–0.2/1.3/0.6
	150 rpm	88.7/–0.2/1.9/2.4	89.2/–0.3/1.9/2.0	89.3/–0.2/1.6/1.8	89.2/–0.2/1.9/1.9	89.4/–0.2/2.0/1.8
	200 rpm	89.8/–0.3/1.4/1.2	88.4/–0.3/2.3/2.9	87.7/–0.5/3.3/4.0	89.3/–0.2/1.7/1.8	89.4/–0.3/2.2/1.9
	250 rpm	89.3/–0.5/2.1/2.0	88.3/–0.3/2.3/3.0	88.5/–0.6/2.9/3.1	88.9/–0.2/2.9/2.3	89.3/–0.5/2.6/2.3
**3260HP**	**Screw Rotational Speed**	**Temperature**				
		**180 °C**	**200 °C**	**220 °C**	**240 °C**	**260 °C**
	50 rpm	89.8/–0.2/1.0/1.7	89.7/–0.3/1.5/1.9	90.2/–0.3/1.2/1.3	90.0/–0.4/1.7/1.7	90.2/–0.3/1.6/1.4
	100 rpm	89.4/–0.2/1.8/2.2	89.6/–0.3/1.4/2.0	89.6/–0.3/1.4/2.0	90.0/–0.3/1.4/1.6	89.2/–0.4/2.5/2.7
	150 rpm	89.7/–0.3/1.5/1.9	89.1/–0.4/2.0/2.7	90.2/–0.2/0.9/1.3	89.0/–0.4/2.2/2.8	90.1/–0.3/1.4/1.5
	200 rpm	89.6/–0.3/1.4/2.0	89.1/–0.3/1.6/2.5	89.4/–0.3/1.6/2.2	89.2/–0.4/2.3/2.7	89.5/–0.5/2.3/2.4
	250 rpm	89.0/–0.3/2.0/2.7	89.2/–0.2/1.0/2.3	89.3/–0.2/1.6/2.3	89.9/–0.3/1.6/1.7	90.3/–0.3/1.5/1.3

Ranges of ΔE according to ISO 2813. 0 < ΔE < 1: invisible color variation; 1 < ΔE < 2: small variations of color, recognizable only by an experienced observer; 2 < ΔE < 3.5: medium variations of color, recognizable by the inexperienced observer; 3.5 < ΔE < 5: distinct color variations; ΔE > 5: large color variations.

**Table 7:** The temperature of 5% and 10% mass loss obtained for PLAs processed with various processing conditions.

T_5%_ (°C)/T_10%_ (°C)						
**2500HP**	**Screw Rotational Speed**	**Temperature**				
		**180 °C**	**200 °C**	**220 °C**	**240 °C**	**260 °C**
	50 rpm	328.0/335.6	304.2/314.5	324.9/333.9	326.6/334.9	323.3/332.8
	100 rpm	329.2/337.0	329.5/337.3	329.2/336.9	328.5/336.5	327.9/336.8
	150 rpm	328.1/335.5	326.9/334.5	328.8/336.2	329.7/337.6	328.6/337.0
	200 rpm	328.4/335.8	329.3/337.5	326.5/333.5	327.8/335.8	326.6/335.3
	250 rpm	330.0/337.8	329.1/336.7	325.8/333.0	328.1/336.5	328.0/335.5
**4032D**	**Screw Rotational Speed**	**Temperature**				
		**180 °C**	**200 °C**	**220 °C**	**240 °C**	**260 °C**
	50 rpm	294.2/301.5	326.7/334.3	305.8/314.6	324.7/332.9	294.9/308.3
	100 rpm	323.7/331.8	297.4/306.7	315.1/324.4	321.2/330.6	296.1/307.6
	150 rpm	324.7/333.1	330.9/338.5	325.4/333.8	324.7/333.0	310.4/320.2
	200 rpm	319.1/328.8	327.6/335.0	328.8/336.1	323.2/330.8	329.8/337.4
	250 rpm	319.5/328.7	312.4/318.7	327.0/334.9	330.1/337.4	304.6/315.8
**3100HP**	**Screw Rotational Speed**	**Temperature**				
		**180 °C**	**200 °C**	**220 °C**	**240 °C**	**260 °C**
	50 rpm	323.2/332.7	328.1/336.2	327.3/335.4	327.4/336.0	299.6/313.8
	100 rpm	311.4/320.8	328.4/336.2	326.6/335.5	308.4/318.4	297.9/311.5
	150 rpm	288.0/300.2	298.7/310.0	327.6/336.2	327.3/335.2	327.4/335.9
	200 rpm	325.7/333.7	321.0/330.1	328.2/336.4	327.7/336.2	327.1/335.9
	250 rpm	328.1/336.1	326.9/335.3	327.2/335.0	326.8/334.4	329.0/335.9
**3260HP**	**Screw Rotational Speed**	**Temperature**				
		**180 °C**	**200 °C**	**220 °C**	**240 °C**	**260 °C**
	50 rpm	325.4/333.7	325.3/334.4	317.5/328.5	328.1/336.2	297.1/312.4
	100 rpm	326.8/335.0	326.6/335.1	326.8/335.7	326.2/334.9	324.0/333.1
	150 rpm	325.8/334.5	327.8/336.3	325.9/334.6	325.6/335.0	320.7/330.6
	200 rpm	323.5/331.6	327.8/336.6	325.1/334.1	325.3/334.4	278.2/289.8
	250 rpm	327.4/336.1	319.5/329.7	327.3/335.7	325.6/333.0	326.9/335.4
